# Extraction of Bioactive Compounds From *Centella asiatica* and Enlightenment of Its Utilization Into Food Packaging: A Review

**DOI:** 10.1155/2024/1249553

**Published:** 2024-09-26

**Authors:** Athira R. S. Pillai, Yuvraj Khasherao Bhosale, Swarup Roy

**Affiliations:** ^1^ Department of Food Technology and Nutrition School of Agriculture Lovely Professional University 144411, Phagwara, Punjab, India; ^2^ Agricultural and Food Engineering Department Indian Institute of Technology Kharagpur 721302, Kharagpur, West Bengal, India

**Keywords:** antioxidant and antimicrobial, biopolymers, *Centella asiatica*, novel extraction, packaging, terpenoids

## Abstract

*Centella asiatica* is a medicinal herb, well known for its phytochemical activities because of the presence of terpenoids and polyphenols, which contribute to the bioactivity of herb extract that can be effectively utilized in the packaging industry. Biopolymers infused with *C. asiatica* extract could be a promising solution in the food sector. The antibacterial and antioxidant qualities of *C. asiatica* can help preserve the quality and lengthen the freshness of food products, thereby preventing food loss. Selection of a suitable extraction method is essential to retain the yield and properties of the bioactive compounds of *C. asiatica* extract. Many research has been conducted on the separation of *C. asiatica* by using conventional and novel extraction techniques and its execution in packaging as a functional component. This review provides an overview of the extraction of phytochemicals from *C. asiatica* and its utilization in biopolymer film as an active component to modify the packaging film characteristics.

## 1. Introduction

Packaging is a crucial component in the food industry to ensure food quality and safety which is commonly dominated by synthetic polymers [1]. Petroleum-based polymers such as polyethylene, polypropylene, and terephthalates are widely used polymers that exhibit high tensile strength and a barrier to light, oxygen, and water vapor which directly influence food quality inside the package [2]. Fossil fuel plastics discarded into the surroundings persist in the ecosystem as microplastics [3]. Microplastics are tiny polymers less than 5 mm in size. These plastics which are discarded into the surroundings can contaminate air, water bodies, and soil and have adverse effects on living beings through bioaccumulation [4]. Advancements in awareness of concerns related to the environment have put packaging under strong criticism because it is an incessant provenance of enormous plastic waste, necessitating extensive research into renewable alternatives [5]. Biopolymers are natural polymers composed of repeating monomer units linked to one another by covalent bonds that are biodegradable and obtained from sustainable or agricultural waste and ecofriendly in nature. Therefore, it can be widely exploited in the packaging industry by replacing conventional polymers [6]. Global biopolymer manufacture was 300,000 tonnes in 2009, rose to 2.11 million tonnes in 2019, and will exceed 2.42 million tonnes by the end of 2030 [7]. Additionally, incorporating phytochemicals from herbs to synthesize packaging film and their sustained release into the food system raises the importance of active packaging. These bioactive compounds can upgrade the structural and functional properties of the packaging film [8].

Biopolymers blended with medicinal extract proved high physical–mechanical and barrier properties and acted as carriers of natural radical scavengers and antimicrobial compounds, modifying the characteristics of the film and increasing its prospective utilization in food packaging. Herbs that contain ample secondary metabolites, antioxidants, and antimicrobials can be exploited in active packaging to a large extent by replacing toxic chemical compounds [9]. Besides, the bioactive compounds released into the food system can benefit the consumer and maintain the oxidative stability of the food components. However, it is important to ensure the controlled delivery of the substance to prevent unforeseen effects on taste and safety. Consequently, *Centella asiatica* extract, with abundant bioactive elements and functional properties, can be included in packaging film.

*Centella asiatica*, widely recognized as Indian pennywort or gotukola, is a medicinal herb belonging to the family Apiaceae. It is native to India, China, Thailand, and Malaysia; widely grown in temperate and tropical wetlands; and well suited for clay or marshy soil [10]. The plant part consists of slender stems, rounded leaves, and rhizomatous rootstock slightly aromatic prostrate creeper of 10–15 cm average height [11]. *Centella asiatica* was recognized in Ayurveda almost 2000 years ago because of its medicinal application. *Centella asiatica* has been used for treating diabetics, Alzheimer's, wound healing, skin allergies, and nerve disease minerals [12]. The herb is a rich source of numerous bioactive substances which include triterpenoids, polyphenols, flavonoids, vitamins, and minerals [12]. Asiaticoside, madecassoside, and asiatic acid are the major compounds recognized in *Centella asiatica* [13]. Moreover, *Centella asiatica* extract exhibits high scavenging activity and antimicrobial properties, which highlight it as a potential functional filler in packaging material [14]. The presence of terpenoids and phenols in *Centella asiatica* acts against food-borne pathogens by disrupting the cellular membrane and inhibiting the wide spectrum of microorganisms [15]. Therefore, extracts of *Centella asiatica* herbs can be exploited in the food industry by adding them as an active component to packaging film [16]. Along with this, the extract can modify the desired properties of the film in terms of durability, shelf life, and technofunctional properties. For performing these functions, the selection of an appropriate extraction method is important to obtain an effective extract with a high yield of phytochemicals.

Active compounds present in the herb can be effectively retrieved from *Centella asiatica* by using conventional and nonconventional extraction techniques [17]. The major obstacles of conventional extraction methods like Soxhlet and cold methods are prolonged extraction time, less efficiency, and the necessity of excess amounts of solvent and compound degradation [18]. The use of novel extraction methods including ultrasonic extraction, microwave, supercritical fluid extraction, radio frequency, and enzyme-assisted extraction techniques can enhance the efficiency of *Centella asiatica* extract and minimize the time of extraction [19]. Modern extraction techniques are mainly designed with sustainability, and they often use minimal amounts of organic solvents and reduce energy consumption, typically yielding high concentrations of desired compounds quickly. Conventional extraction methods like Soxhlet and heat reflux can cause a threat to the phytoconstituents through degradation by excessive heating and result in lower yield. Methods such as ultrasound and microwave can extract the substance in minutes rather than hours or days to promote their application at the industrial level. Furthermore, the novel extraction techniques allow for greater selectivity for specific compounds which is an added advantage. Furthermore, the nutritional and functional quality of the material is preserved due to the controlled extraction condition [20].

Thus, this review is aimed at investigating various extraction methods for obtaining bioactive compounds from *Centella asiatica* leaves and evaluating the potential application of the extract in packaging film. Novel extraction methods such as ultrasound, microwave, and supercritical fluid extraction improve the efficiency in the yield of bioactive compounds such as triterpenes, polyphenols, and flavonoids compared to conventional extraction methods and their contribution to the phytochemical activities such as antioxidants and antimicrobials. Furthermore, the study focuses on the characteristics of the packaging film incorporated with the extract. In summary, the review explores the effect of optimization of extraction techniques for the extraction of phytochemicals of *Centella asiatica* and the mechanism of the active substance in the packaging film for improving barrier, mechanical, and functional properties. The insights of this review are expected to bring more interest in functional herbs including sustainable and smart packaging.

## 2. Extraction of Bioactive Compounds From *Centella asiatica*

Extraction is the method of isolation of phytochemicals from the plant part. The research studies proved *Centella asiatica* contains ample secondary metabolites, majorly triterpenoids and phenolic compounds, which contribute to the therapeutical application of the herb and other functional properties [21]. The selection of a suitable extraction method is very important to increase the yield of extract and the immensity of the bioactivity [22]. In addition, the choice of appropriate solvent is also an essential component to increasing the efficiency of the extract of *Centella asiatica*, as different solvents have varying affinities towards different compounds [23]. Polar solvents are suitable for polar compounds, and nonpolar extracts out nonpolar compounds from the herb. Furthermore, the stability of the specified components and the toxicity of the solvent are essential to ensuring an effective and safe extraction process [24]. Currently, there are studies conducted on the use of green solvents for extracting bioactives from plant material. The study conducted by [25] successfully extracted asiaticoside from *Centella asiatica* using triethylammonium sulfate as solvent. Triethylammonium is an ionic liquid synthesized through an acid–base neutralization reaction. The solvent is nonvolatile, thermally stable, and biodegradable and can be used as a better substitute for organic solvent extraction. Along with solvent, temperature, frequency, time, and power are also critical parameters to be considered when extracting the herb. To further improve the extraction efficiency, pretreatment can be applied to the sample. Pretreating samples before extraction is a technique used to improve extraction efficiency by modifying the physical and chemical properties of the material, making the target compounds more accessible. Among the various pretreatment methods, drying is widely used to preserve the nutritional quality of medicinal herbs. However, drying techniques, such as sun drying, vacuum drying, or tray drying, are typically energy-intensive and time-consuming, and nonthermal pretreatments like cold-plasma treatment are more efficient and energy-saving [26]. Recent studies have shown that using cold plasma as a water pretreatment can effectively maintain the functional properties of *Centella asiatica*, offering a promising alternative to traditional methods by retaining the herb's beneficial qualities while potentially reducing energy consumption and processing time [27]. Different extraction methods and their effect on *Centella asiatica* are mentioned in Table 1.

Prolonged extraction duration and elevated temperature degrade the bioactive elements in *Centella asiatica*, thereby reducing the calibre of the extract. Recent studies on the adoption of novel extraction techniques such as ultrasound, microwave, and supercritical fluid extraction of *Centella asiatica* prove the advantages over traditional extraction methods of herbs [35]. Nonconventional extraction methods lower the quantity of solvent required and reduce the time and temperature compared to conventional techniques and can be considered for the effective extraction of *Centella asiatica* [36].

### 2.1. Ultrasound Extraction

**Figure 1:**
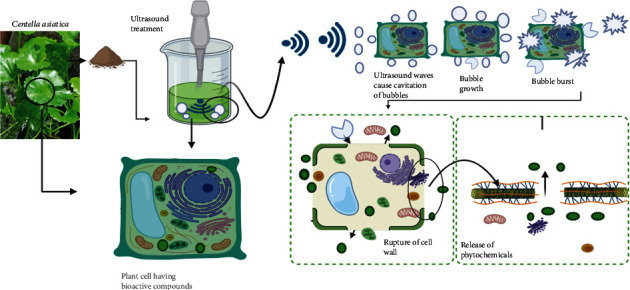
Mechanism of ultrasound-assisted extraction of *Centella asiatica*.

Ultrasound-assisted extraction (UAE) is a proven green technology due to its high extraction efficiency and ability to lower the time and cost. The technique uses ultrasound energy to create cavitation bubbles. Breakdown of the bubbles generates microjet turbulence, which disrupts the plant membrane, which increases the solute penetration into the solvent and leaks out of bioactive [37]. The mechanism of the ultrasound extraction process is schematically shown in Figure 1. The increased mass transfer rate aids in the reduction of extraction time. Moreover, ultrasound extraction requires minimal solvent and reduces heat-sensitive compound degradation. In comparison to traditional techniques, ultrasound extraction is thought to be highly convenient and energy-efficient. The method is one of the best methods for obtaining a high yield of phenolic and flavonoids with high phytochemical properties like antioxidant, anti-inflammatory, and antimicrobial [28]. Application of frequency above 20 kHz causes the degradation of bioactive compounds, so it is not recommended. The method requires less time for extraction and solvent usage, and a reduced sample size is required compared to conventional methods. It is extremely efficient and accounts for minimal compound loss due to the use of controlled temperatures.

Recent research [38] reported that UAE provides a significant yield of secondary metabolites in *Centella asiatica*. Ultrasound treatment of 200 W for 20 min gave a better yield of total polyphenol value of 2255.21 ± 33.35 GAE mg/100 g DW, and 10 min of treatment gave total flavonoid contents of 2219.80 ± 5.39 CE mg/100 g DW. The enhancement could be due to the stimulation of shikimic acid–phenylpropanoid metabolism, which results in the biosynthesis of flavonoids and phenolics and accumulation. There is a significant increase in antioxidant activity observed in ultrasound-treated leaves of *Centella asiatica* compared to untreated leaves [38]. The application of ultrasound to *Centella asiatica* increases the demand for peroxidase and catalase to decompose hydrogen peroxide (H_2_O_2_), protect plant cells from oxidative damage, and boost the activity of phenylalanine ammonia-lyase (PAL) and transaldolase (TAL) to synthesize flavonoids and phenolics. Peroxidase and catalase are stress marker enzymes that help with free radical scavenging activity. TAL and PAL, which are involved in the activation of the phenylpropanoid pathway for phenolic biosynthesis, are important plant resistance markers. L-Tyrosine is converted to *p*-coumaric acid by TAL, and L-phenylalanine is transformed to *trans*-cinnamic acid by PAL. At 10 min, the most significant peroxidase and catalase activity was noted, and it is observed that, compared to untreated leaves, the enzyme activity of TAL and PAL in *Centella asiatica* leaves were increased by 1.30- and 1.55-fold, respectively, after 10 min of ultrasound treatment [38].

Another study [39] indicated that UAE is the better method of extraction compared to Soxhlet and maceration and obtained a higher yield of terpenoids (0.34%) and flavonoids (0.11%). Selection of the appropriate solvent is one of the critical factors that determines the yield of the extract. According to [40], a binary solvent of ethanol and water in a 40:60 ratio gave a better yield than individual solvents. However, using conventional solvents like ethanol and methanol has some drawbacks such as toxicity, flammability, and nonbiodegradability. Therefore, solvents that are derived from natural resources can be replaced instead of conventional organic solvents. Natural deep eutectic solvent (NADES) is used successfully for extracting *Centella asiatica* and gives a higher yield of bioactive compounds. Ultrasound extraction of asiaticoside from *Centella asiatica* using betaine-based NADES was developed, and the highly efficient solvent was betaine–levulinic acid at a 1:2 molar ratio and a water content of 30% (*w*/*w*). The optimum extraction conditions were 32 min, 36°C temperature, 140 W ultrasound power, and 49 mL/g L/S ratio, yielding an extraction yield of 229.92 ± 1.67 mg/g. The study found that the optimized UAE-NADES technique yielded more than the traditional method of extraction [28].

### 2.2. Microwave-Assisted Extraction (MAE)

**Figure 2:**
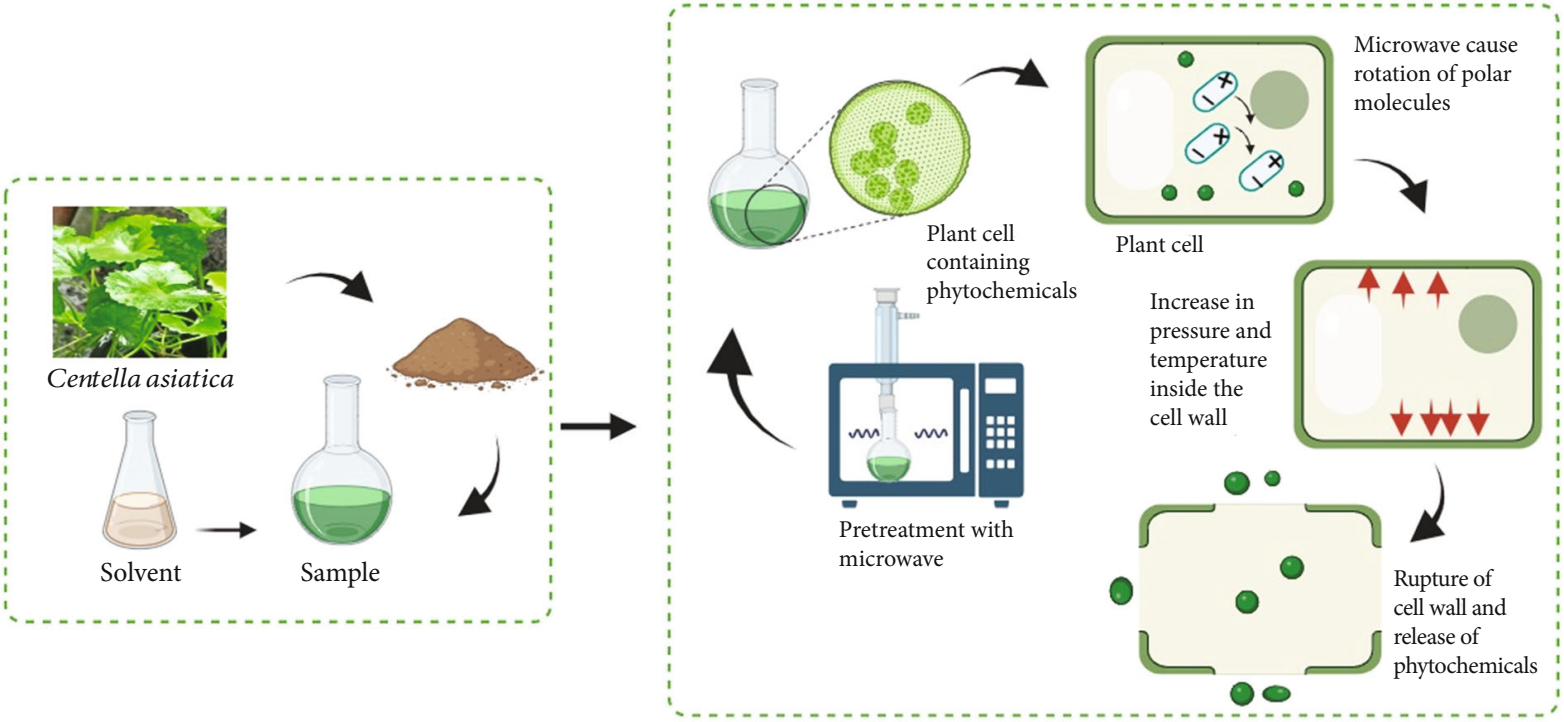
Mechanism of microwave-assisted extraction of *Centella asiatica.*

MAE has gained popularity as a novel method for extracting bioactives from different sources using microwave radiation. This methodology has high yield efficiency and requires less energy and time compared to traditional extraction techniques. Microwave radiation heats the moisture inside plant cells, causing evaporation and high cell wall pressure. This high pressure bursts the cell membrane, and the compounds exudate through the ruptured cell wall [41]. The mechanism of MAE is illustrated in Figure 2. The selection of relevant solvent has a direct effect on the extraction yield. According to the study conducted by Rahmawati et al. in 2021 [32], the yield of the phenolic compound obtained by solvent-free extraction microwave extraction is 2.39869 mg GAE/g, comparatively much less than the studies that used solvent. A mixture of solvent and water has a significant effect on extraction, and polar solvents are highly suitable for the extraction of polyphenols and terpenoids which contain hydroxyl groups, which makes them polar [32]. Solvents such as ethanol and methanol which are polar in nature interact with the functional group present in the bioactive compounds and break the bonds that keep them bound with the plant tissue.

Selection and concentration of the solvent, liquid-to-solid ratio, irradiation time, and microwave power are important for the isolation of the targeted compounds [42]. Using methanol as a solvent in microwave extraction showed the highest yield of bioactive compounds such as asiaticoside, madecassoside, madecassic acid, asiatic acid, kaempferol, quercetin, rutin, and chlorogenic acid. Methanol is the most efficient solvent for the extraction due to its polarity. The study shows that the extraction efficiency increases with the polarity of the solvent [24]. Followed by methanol, a mixture of ethanol and a concentration of 50% water (*v*/*v*) enhanced the solubility of terpenes [32]. Extraction by using microwave and methanol as solvents at 800 W micropower for three extraction cycles of 2 min irradiation time resulted in maximum antioxidant activity. As the yield of phenolic compounds increased, antioxidant activity also increased [24].

According to [43], for the comparison of MAE under vacuum and atmospheric conditions, phytochemicals from dried leaves and fresh leaves of *Centella asiatica* showed that applying 60 kPa by vacuum microwave extraction resulted in a higher extraction rate of terpene saponins from fresh leaves, but in the case of dried leaves, the atmospheric condition showed higher terpene and total phenolic compounds. Using high temperatures and the contact between oxygen and material can affect the heat-sensible bioactive compounds present in *Centella asiatica*. The application of vacuum MAE resulted in the separation of triterpenoids in higher concentrations from fresh leaves than MAE due to vacuum pressure. In accordance with dried leaves, maximum triterpene and total phenol content were obtained by MAE due to the influence of temperature which affected the diffusivity of the compounds into the solvent [43]. Another factor that affected the yield is microwave power. Application of power above 500 W decreased the yield. Maximum yield is obtained at the range of 300 W. Increasing the extraction time and microwave power significantly affected the yield obtained. Using 300 W for 15 min increased the evaporation rate which contributed to the higher yield. However, using power beyond 500 W decreases the yield by variation in temperature variation leading to the thermal degradation of compounds, and it is not recommended [31].

A significant rise in microwave power leads the electromagnetic energy to be transmitted to the plant matrix more quickly, and this leads to the development of internal heat stress, which breaks down the cellulose content of the cell wall and creates fractures that make analytes easily evacuate out [20]. Up to a certain extent, an increase in irradiation duration likewise improved yield when microwave power increased. Even with greater microwave powers, there is not enough irradiation time to induce thermal stress within the plant matrix, which means that the impact on cell wall integrity is not severe enough to result in rapid and simple analyte leaching. However, increased microwave power combined with a longer extraction period may result in significant heat stress inside the matrix of the plant and within the solvent, which will degrade the analytes [24].

### 2.3. Supercritical Fluid Extraction

**Figure 3:**
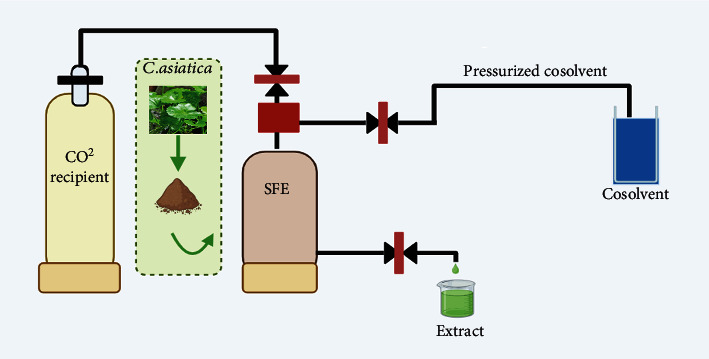
Supercritical-fluid extraction of *Centella asiatica*.

Supercritical fluid extraction is a widely applied modern method that is used as a substitute for the conventional method of extraction due to its selectivity of compounds, being less time-consuming, nontoxic, and environment-friendly. The supercritical fluid shows low viscosity and a better-flowing property like gas at a critical point [44]. CO_2_ is the widely used gas in the supercritical method due to its minimal critical pressure, and temperature as well as CO_2_ increases the solubility of nonpolar compounds, and using ethanol as a solvent enhances the extraction of nonpolar compounds. The application of high pressure can also enhance solubility [34]. The supercritical fluid extraction method applied to *Centella asiatica* increased the percentage yield of major bioactive compounds asiaticoside, which shows antioxidant, antimicrobial, and anti-inflammatory properties [33]. The extraction of *Centella asiatica* using supercritical fluid extraction is illustrated in Figure 3.

According to the study on the impact of variables such as cosolvent pressure and temperature, the application of pressure has a high effect on the yield of asiaticoside. When *Centella asiatica* was extracted at a temperature of 80°C with 10% cosolvent at 10 MPa pressure, the yield rose from 0.6102 to 0.6365 *w*/*w*. The solubility of the solvent increased because of the density of the fluid, which is directly proportional to the applied pressure, which results in a high yield [33]. Increasing the temperature above 80°C and the cosolvent percentage by more than 10% has no significant enhancement in the asiaticoside quantity, and it may cause the plant tissue to destroy and discharge the least quantity of compounds. The extraction yield of asiatic acid and asiatic acid obtained through the subcritical water extraction method increased with an increase in temperature, but there were no observable changes concerning pressure. A higher yield is obtained at the optimum condition of 250°C and 40 MPa, that is, 78 mg/g asiatic acid and 10 mg/g asiaticoside, which is higher than the extraction using organic solvents ethanol and methanol at room temperature but not as high as its boiling temperature [45]. However, in a separate study by [44], pressure affected the extraction of asiaticoside concentration but had no observable effect on total phenolic content compared to maceration.

## 3. *Centella asiatica* Extract Incorporated Packaging Film

Food packaging is defined as a physical obstacle to protect food from deterioration and ensure the food quality and shelf life. Efficient packaging can safeguard the food from microbes, moisture, UV light, and oxygen, which are responsible for food spoilage [15]. The utilization of synthetic packaging materials, which are nondegradable, can create various environmental hazards [46]. Replacing synthetic polymers with plant-based substances is a promising field in food science due to sustainability and economic efficiency. As a concern about the environmental impact created by synthetic polymers, naturally obtained polymers including starch, zein, chitin, cellulose, and gelatin have acquired more popularity in the food sector due to their biodegradability and advantages over petroleum-based polymers [47]. Furthermore, the addition of functional compounds from plant sources such as essential oils, polyphenols, flavonoids, antimicrobials, and antioxidants can improve the physical, barrier, and structural properties of the biopolymer and can be widely exploited in food packaging [48].

Biopolymer combined with bioactive substances from herbs can be utilized to prepare active packaging film. The addition of the bioactive substance can provide multiple functionalities to the film by protecting the food from deterioration from microbes and increasing the shelf life due to antioxidants and antimicrobials [49]. Active packaging is a novel technique that provides an alternative to conventional packaging. Phytochemicals present in the extract can act as natural antioxidants and antimicrobial agents [50]. Added functional compounds in packaging film interact with the food components and provide several health benefits as well to the consumer. In addition, the active substance acts as a hindrance to oxygen, moisture, water vapor, and microbes, thereby protecting the food from degradation [51]. Most of the current research has shown that the incorporation of natural additives into the packaging material can imply some beneficial effects on the characteristics of the packaging substance as well as minimize the ecological impact [52]. *Centella asiatica*, the herb with inherent medicinal and functional properties, includes antioxidant, antimicrobial, anticancer, and anti-inflammatory properties, highlighting its potential application as an active substance in packaging film.

**Figure 4:**
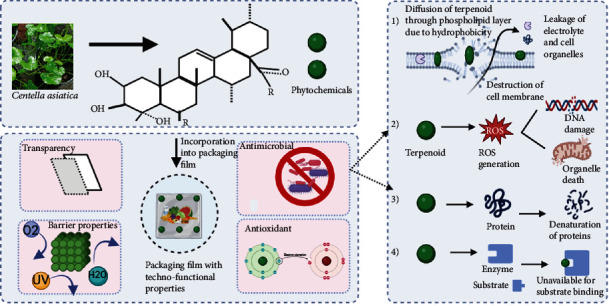
Development of functional packaging film incorporated with *Centella asiatica* extract.

*Centella asiatica* is rich in ample bioactive compounds. Green extraction of gotukola retained its phytochemical properties, and no degradation of compounds was observed. It is successfully shown that the extract derived from the herb contains higher concentrations of phenolic compounds, terpenoids, and flavonoids, which can act to impart antioxidant and antimicrobial properties. Oxidation and microbes are the major reasons for food deterioration [9]. There is rapid growth in the manufacture of antimicrobial and antioxidant packaging film from natural sources, which does not cause damage to living beings, and the bioactive compounds provide benefits to humans and improve the technofunctional properties of the packaging film [53]. Incorporation of *Centella asiatica* extract in small quantities into the packaging film can prevent microbial contamination and prevent oxidational damage to the food. According to different literature, the extent to which technofunctional properties change is determined by the bioactive compound present and the amount of herb extract immersed into the film (Table 2). In the case of *Centella asiatica*, the major compound terpenoids directly influence the properties of the biopolymer (Figure 4).

The preparation of packaging film involves various methods; solvent casting is the wide method used for the fabrication of *Centella asiatica* extract-infused film, as it is the easy method of preparation and provides a quality thin film with improved properties. The method of preparation includes dissolving the selected biopolymer in a suitable solvent, spreading it into a surface, and allowing it to leave a solid cast by removing the solvent [57]. However, the solvent casting method requires a long time for processing, and it is preferred for laboratory scale-up only, not for large-scale production in industrial applications. Therefore, extrusion and blowing methods are commonly used for the preparation of packaging film under industrial applications [58]. The structure and function of the biopolymer are characterized by the chemical and physical interaction between the polymer film and the herb extract. The polymer and extract's nature, chemical properties, concentration, conformational flexibility, strength, and stereochemistry of the active substance contribute to the nature of the interaction [59].

### 3.1. Effect of *Centella asiatica* Extract on Various Properties of the Film

#### 3.1.1. Mechanical Properties

Tensile strength, young module, and elongation are the major parameters to represent the amount of load that can withstand the film, and elongation resistance defines the film capacity during stretching to protect food material intact. Based on the added concentration and phytochemistry of the extract, the properties of the film can be improved [60]. Structure and functionality can be affected because of the chemical and physical interaction between the polymer and the added substance. A linkage is formed between the hydrogen bonds of phenolic compounds and the molecules in the polymer film, resulting in intermolecular interaction, and this interaction could affect the characteristics of the packaging film [9].

The tensile strength can be defined as the pressure that the film can withstand during handling and transportation without any damage [56]. The addition of *Centella asiatica* extract into the biopolymer film enhanced the tensile strength. According to the study conducted by [54], the properties of the gelatin film incorporated with the *Centella asiatica* herb extract showed better mechanical properties compared to the film without the extract. The modification of the mechanical strength of the packaging film could be through the formation of hydrogen bonds. The hydroxyl groups of the phenolic compounds present in the extract interact with the amine groups of gelatin molecules, leading to the hydrophobic interaction and hydrogen bonding within the film matrix. This interaction strengthens the overall structure of the film, resulting in increased tensile strength values compared to control films without the extract. The addition of *Centella asiatica* extract facilitates cross-linking reactions between the extract and the gelatin polymer matrix. This cross-linking reduces the mobility of the biopolymer chains, which contributes to a higher melting point and improved mechanical stability of the film. Specifically, studies have shown that films with added *Centella asiatica* exhibit higher melting temperatures, indicating a more stable and robust film structure. Fish gelatin films contain a linear structure that promotes greater cross-linking with phenolic compounds [61].

A concentration of 25% extract expressed a result of 2.15 MPa compared to the control which resulted in 0.85 MPa. But in the case of film developed using silver nanocolloid synthesized from *Centella asiatica*, it expressed a gradual reduction in tensile strength with an increase with silver nanoclusters. This is due to the reduction of cohesion of the polymer matrix affected by the incorporation of silver particles [62]. At the same time, enhancement in the concentration of extract showed higher elongation at break (Eab) value. There are numerous reasons for the reduction in tensile strength and rise in elongation at packaging film breakage associated with a higher concentration of silver nanoparticles derived from *Centella asiatica*. Firstly, integrating silver nanoparticles could cause defects in structure or irregularities in the film matrix, which would lower its overall tensile strength. Additionally, the polymer-filler interaction may change when nanoparticles are present, which could result in weakened intermolecular forces and reduced mechanical strength [63].

The enhanced ductility and flexibility brought about by the existence of silver nanoparticles may be the cause of the increase in Eab. While the film completely breaks, these nanoparticles may serve as stress concentrators, encouraging localized deformation and elongation. Additionally, the flexibility of the film matrix may be impacted by the dispersion and distribution of silver nanoparticles within it [63]. The addition of extract might alter the film structure and enhance the gelatin length, resulting in a gradual upsurge in Eab. However, another research showed that the 0.3% concentration showed a high Eab value compared to the 0.7% concentration. It was due to the excess extract increasing the pore size of the film and creating a rupture point, which led to the decline in Eab value [55].

#### 3.1.2. Thermal Properties

The thermal property of a packaging film determines the conductivity of heat, or in another way, it can be said that how the film reacts with heat fluctuations. Thermogravimetry and differential scanning calorimetry are the methods used to measure the thermal property [64]. The inclusion of *Centella asiatica* extract into the gelatin film for the synthesis of packaging film influenced the thermal properties of the material. There was an advancement in thermal stability observed by adding the plant extract to the film. The addition of a concentration of 25% extract showed a higher melting point (65.29°C) than 5% extract (59.12°C). *Centella asiatica* extracts in packaging film may have a greater degree of thermal stability due to the presence of heat-resistant compounds [54].

Polyphenols, triterpenoids, asiaticoside, and madecassoside have been identified in *Centella asiatica* to endure high temperatures without deterioration, making the packaging film more resistant to heat exposure [65]. The chemical composition and molecular structure of *Centella asiatica* extract are likely to contribute to the packaging film's thermal stability. By interacting with biopolymers through various mechanisms, terpenoids can modify the thermal stability of packaging films. The biopolymer matrix is strengthened by hydrogen bonds or other noncovalent interactions that they form. Triterpenoids can form hydrogen bonds and interact with biopolymers such as gelatin. The structural integrity of the film is increased due to the cross-linking, making it more resistant to thermal degradation. The presence of these compounds can stabilize the polymer chains against thermal stress, thereby probably raising the melting temperature and improving overall thermal stability [66].

Terpenoids can also have plasticizing properties that increase elasticity and reduce the possibility of thermal deterioration. The particular interactions affect the packaging film's overall thermal performance and are dependent on the terpenoid and biopolymer involved [66]. Polyphenols also contribute to the increase in the melting point value due to the phenolic binding with the gelatin, resulting in hydrogen and covalent bond formation [54]. Furthermore, the blending of extract into the film promotes cross-linking and enhances thermal stability, resulting from a higher activation energy. Higher thermal stability was observed in the case of nanocomposite film prepared using *Centella asiatica.* This was due to the properties of zinc oxide nanoparticles, which have heat insulation properties and can enhance interaction between polymer chains and prevent the escape of volatile compounds [56].

#### 3.1.3. Water Vapor Permeability

The permeability of water vapor through packaging substances is a crucial parameter that affects the quality of food items. The amount of water contained can stimulate microbial growth, thereby causing food deterioration, and the exchange of water vapor inside the food and outside the environment can also influence food quality. Entry of water into the dry food can also cause spoilage [67]. That is why a good packaging material should have less permeability for water vapor. The inclusion of the *Centella asiatica* extract limits the water vapor permeability by reducing the free space in the polymer matrix through the emergence of a dense network structure by the interaction of biopolymer and polyphenol elements. Increased concentration of the extracted results in the fabrication of a tortuosity path, which decreases the permeability of water molecules across the film [68].

A comparable result was noted in the chitosan–gelatin film incorporated with Chinese hawthorn [69] and the biodegradable film prepared using grape seed extract [70]. This proves that plant-based extract contains high polyphenols, and its concentration has a direct impact on the film permeability of water vapor. An increased quantity of the extract gradually decreased the water vapor permeability in the film. The film containing 0.7% *Centella asiatica* extract resulted in 1.13 g.m/m^2^ s Pa, and 0.3% extract showed 1.11 g.m/m^2^ s and 1.03 g.m/m^2^ s Pa for control, that is, without extract. Hence, the inclusion of *Centella asiatica* extract enhanced the physical characteristics of gelatin films and caused the films to limit the permeability of water vapor.

#### 3.1.4. Light Transmission

The transparency of a film is a crucial factor because it contributes to the acceptance of the consumer since it allows one to examine the food's freshness. On the other hand, light transmission is another critical barrier property to enhance the food shelf life. Light can lead to the decomposition of nutrients in the food, particularly with lipids, and result in the development of off-flavour. Therefore, packaging film should be highly resistant to both visible light and ultraviolet rays [71]. Gelatin film added with extract 0.3% and 0.7% shows low light transmission compared to the control film, which signifies a better barrier to UV light. The polymer's alignment or arrangement within the film most likely determined how much light could pass through it. Modifications in the transparent material's composition can lead to considerable adjustments in its optical characteristics. The uniform dispersion of *Centella asiatica* in the packaging film matrix creates a light-scattering effect that prevents the penetration of light through the packaging film. The bioactive compounds present in the herb extract, such as phenolic compounds and triterpenoids, interact with the polymer chains, altering the alignment and arrangements of the film structure. This change in the film's microstructure leads to a decrease in light transmission and also absorption of UV light [55].

In addition, *Centella asiatica* improved the transparency of the packaging material. *Centella asiatica*, which is hydrophobic in nature, affects the transparency of packaging material. Triterpenoid substances found in *Centella asiatica*, such as madecassoside and asiaticoside, are responsible for the plant's hydrophobic properties [72]. These hydrophobic substances can repel water molecules when added to packaging films, preventing the molecules from being absorbed into the film's structure. Water absorption frequently causes packaging materials to expand and lowers some of their clarity. This phenomenon can be described chemically by the certainty that triterpenoids are nonpolar compounds. Water, which is polar in nature, interacts with nonpolar substances less favourably, which lowers its affinity and decreases its absorption of water. Therefore, reducing the effects of water-induced structural deterioration helps retain its transparency [55].

#### 3.1.5. Antioxidant Property

Antioxidants are substances that occur naturally or are synthesized chemically and can hinder the action of free radicals that are formed through the oxidation reaction of oil and fat in food or via oxidation stress in human beings [73]. Oxidative reactions primarily target lipids, particularly polyunsaturated fatty acids (PUFAs), which is a major problem for both natural and processed foods. Though less so, oxidative reactions can also have an impact on proteins and carbohydrates [74]. Food oxidation resulting from modified lipids and proteins produces unpleasant odours, tastes, colours, and textures. These changes not only impair the nutritional value and sensory appeal of food items but also threaten consumer safety by generating potentially hazardous compounds [75]. Nowadays, the synthesis of packaging film with radical scavenging activity by incorporating natural antioxidants has become popular due to its numerous benefits. Packaging films containing chemically synthesized antioxidants endure obstacles because consumers have a negative perception of these products [76]. Food manufacturers are under greater pressure as a result to look for safer natural alternatives, like phytochemicals. These packaging films exhibit high protection for food and increase shelf life by preventing oxidation. Extract of *Centella asiatica* possesses high antioxidant activity and contains a broad amount of phytochemicals such as polyphenols and terpenoids with antioxidant effect, considered one of the important properties to consider extract as an ingredient in packaging film as they are capable of scavenging free radicals in food products, aiding in preventing the oxidation of food matrices.

*Centella asiatica* is a major source of phytochemicals. According to the study conducted, among the different medicinal herbs such as *Centella asiatica*, *Euphorbia hirta*, and *Alstonia scholaris*, *Holarrhena antidysenterica*, in 80% methanol, *Centella asiatica* showed a high concentration of polyphenol and antioxidant activity of 88.16%. The existence of phenolic compounds in the extract contributes to the higher free radical scavenging activity [77]. When the extract is combined with the packaging film, the material exhibited excellent antioxidant activity due to the interaction of polyphenol with the gelatin film through covalent interaction. As the quantity of herb extract increased, the antioxidant activity of the film also improved [54]. The film containing 25% herb extract showed an increase in total phenol content of 4.63 mg/g of GAE with antioxidant activity of 47%. The active substances such as polyphenols and terpenoids present in *Centella asiatica* contributed to the antioxidant activity. The aromatic structure and number of hydroxyl groups define the scavenging potential of polyphenols. As the number of hydroxyl groups in the polymeric structure increases, antioxidant potential also increases. Two primary mechanisms are thought to be responsible for the molecular basis of polyphenols' antioxidant activity: either they directly interact with the reactive oxygen species as primary antioxidants or they involve the chelation of free metals as secondary antioxidants. The primary antioxidant function of polyphenols is linked to their ability to neutralize free radicals by the transfer of electrons, thereby stabilizing them. This occurs by interrupting the O-H bond and engaging in a process of hydrogen atom transfer between the phenolic antioxidant (ArOH) and the free radical (R•) [78]. Recent research by [79] has shown that polyphenols, cellular antioxidant enzymes, glutathione peroxidase, catalase, and superoxide dismutase (SOD) enzymes also influence the antioxidant activity of *Centella asiatica* extract. *Centella asiatica* increases the production and function of SOD and glutathione peroxidase [79]. These enzymes are essential for both preventing oxidative damage to cells and neutralizing reactive oxygen species [80]. The antioxidant qualities of *Centella asiatica* are attributed to several bioactive substances, majorly triterpenoids. Active compounds present in *Centella asiatica* have significant interaction with antioxidant enzymes SOD and glutathione peroxidase with asiaticoside binding energy −10.2310 kcal/mol and madecassic acid −10.1232 kcal/mol. Studies have shown that *Centella asiatica* aids in the production of glutathione, an essential antioxidant for glutathione peroxidase to function. Elevated glutathione levels have the potential to augment the activity of glutathione peroxidase to protect it from oxidative damage [81].

#### 3.1.6. Antimicrobial Activity

Food spoilage is the process by which food becomes contaminated, losing nutrients, colour, and texture as well as allowing pathogenic microbes to grow and degrade the product's quality to the extent that it is no longer edible. Microbes are the most critical factor causing food spoilage [82]. A novel invention, the antimicrobial packaging system suppresses the growth of specific microorganisms that contaminate food by encasing an antimicrobial agent in a polymer film. Therefore, packaging film having antimicrobial properties is very important in food preservation to prevent the growth of microbes during transportation and storage [83]. Methods like disc diffusion and total viable colony count are commonly used to measure antimicrobial activity. The inclusion of antimicrobials into the polymer helps to prevent microbes and increase the life span of perishable foods. Antimicrobials obtained from plant extract have become popular in the packaging of food because they are obtained naturally, nonhazardous, and easy to degrade [84]. The high concentration of phenolic compounds in the herbs is attributed to the strong inhibition against certain food-borne pathogens. The efficiency of the antimicrobial properties of the plants depends on the number of single and double alternate bonds and hydroxyl groups in the reactive groups [83].

**Figure 5:**
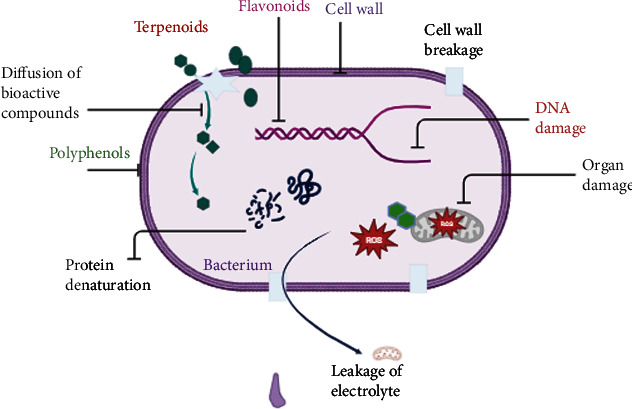
Schematic representation of antimicrobial action mechanism in *Centella asiatica.*

There are several researches conducted on the antimicrobial properties of *Centella asiatica*. *Centella asiatica*, which contains several phytochemicals, including triterpenoids, volatile oils, polyphenols, and flavonoids, which are extracted using aqueous or solvent, contributes to the antimicrobial properties (Figure 5). There are several reasons for antimicrobial action, such as polyphenols can damage microbial cell membranes, allowing cell contents to leak out. Furthermore, polyphenols obstruct essential cellular functions by interfering with DNA and microbial enzymes. By producing reactive oxygen species and oxidative stress in microbial cells, their antioxidant qualities also support antibacterial activity [85]. In contrast, terpenoids, which are lipophilic in nature, pass through the phospholipid layer of the microbial cell wall, thereby disrupting the membrane and leading to the outflow of proteins and enzymes and depletion of ATP [86].

The methanolic extract of *Centella asiatica* exhibited an 11 mm inhibition zone against *Streptococcus pyogenes*, *Staphylococcus aureus*, *Streptococcus faecalis*, and *Escherichia coli*. In different investigations, the extract of *Centella asiatica* using methanol as a solvent demonstrated higher inhibitory activity against various bacteria compared to the solvents ethyl acetate, acetone, and aqueous extracts [87]. The antimicrobial activity of *Centella asiatica* extracts uses different solvents such as methanol, ethanol, hexane, and water against *Bacillus cereus*, *Escherichia coli*, *Salmonella enterica*, *Staphylococcus aureus*, *Aspergillus niger*, *Salmonella typhimurium*, and *Candida albicans* using the agar diffusion method at concentrations 10, 100, 250, and 500 mg/mL. Results discovered that most elevated concentrations of 500 mg/mL of methanolic extract indicated noteworthy inhibition against all the microbes *Bacillus cereus* (13.50 mm), *Escherichia coli* (9 mm), *Staphylococcus aureus* (13.50 mm), *Salmonella typhimurium* (10.67 mm), and *Aspergillus niger* (7.67 mm). *Bacillus cereus* and *Staphylococcus aureus* which are gram-positive bacteria and yeast were found to have a greater inhibition zone than those of the gram-negative bacteria *Escherichia coli*, *Salmonella typhimurium*, and *Aspergillus niger*. Additional outer membrane present in the gram-negative bacteria prevents the permeability of the membrane and penetration of antimicrobials through cell walls [88]. These results indicate the potential effect of *Centella asiatica* against pathogens. Many secondary metabolic compounds with a range of pharmacological characteristics, including antimicrobial ones, were found in *Centella asiatica.*

The major secondary metabolite asiatic acid derived from *Centella asiatica* contributed to its antimicrobial properties [89]. Asiatic acid was found to exhibit potent antibacterial activity against both gram-positive *Enterococcus faecalis* and gram-negative *Escherichia coli*, with minimal inhibitory concentration and minimum bactericidal concentration values of 20, 32, 24, and 36 *μ*g/mL, respectively. This phytochemical inhibited cell motility caused damage to the pathogen's membrane and changed the pathogen's morphological structure [90]. Additionally, *Centella asiatica* is a significant source of essential oil. Extracted volatile oil was used for the microplate dilution method to test the plant's antibacterial properties. The results demonstrated that *Centella asiatica* essential oil had potent antibacterial activity against a variety of bacteria including *Staphylococcus aureus*, *Escherichia coli*, *Bacillus subtilis*, and *Shigella sonnei.* These outcomes demonstrated the essential oil from *Centella asiatica*'s wide-ranging antibacterial properties. Therefore, *Centella asiatica* extract can be added to packaging film to prevent microbial contamination because of its broad range of antimicrobial activity. Rice starch film incorporated with zinc nanoparticles which are synthesized from Indian pennywort presented high inhibition against *Escherichia coli*, *Aspergillus niger*, and *Salmonella typhimurium*. The antibacterial property of the film is because of cell wall interruption of the microbes by zinc ions due to the production of strong oxidizing agent hydrogen peroxide by zinc oxide nanoclusters, which damages the cell membrane of the pathogen [56]. Therefore, it can be concluded that *Centella asiatica* extracts blended film can effectively act against the growth of harmful food pathogens.

## 4. Conclusion

The application of natural substances into packaging film is a promising trend that has been growing recently. *Centella asiatica* contains plenty of phytochemicals that exhibit a broad range of beneficial properties including antimicrobial and antioxidant. Moreover, bioactive compounds present in the extract, primarily terpenoids and polyphenols, are attributed to various functional, mechanical, barrier, and biochemical properties of the packaging film and thereby preserve food for a long time. Presently, the demand for active substances in packaging is increasing due to their functionality and sustainability. Thus, *Centella asiatica* extract can be efficaciously exploited for food packaging. The choice of suitable extraction methods is also important to retain the properties and yield of the compounds. Different studies reported that novel extraction techniques increased the phytochemical properties and prevented the degradation of compounds compared to Soxhlet and cold extraction. Isolated bioactive compounds can be included in packaging applications, which assuredly provide safety to food and aid in preventing hazards to the environment. The natural antioxidants and antimicrobials in the herb can be explored for further utilization, and future studies should focus on integrating *Centella asiatica* extract into food wrapping film synthesis and exploring its probable use in extending shelf life while ensuring safety and compliance with food safety regulations. The huge demand for herbal and natural products is expected to drive market growth for *Centella asiatica* extract in various industries including food, cosmetics, and pharmaceuticals and enhance the scope of research on the optimized extraction methods for large-scale application to save time and energy. In conclusion, the extraction and application of *Centella asiatica* in packaging film hold significant promise for a sustainable future and innovative application in various industries.

## Figures and Tables

**Table 1:** Different extraction methods and their effect on the extract quality of *Centella asiatica.*

**Sample**	**Extraction method**	**Solvent**	**Parameters**	**Compounds extracted**	**Bioactivity**	**Reference**
Dried leaves (40°C + 18 h)	Soxhlet	Methanol	64.7°C	Asiaticoside: 19.93 ± 0.46 mg/g Madecassic acid: 11.68 ± 1.18 mg/g	Antioxidant activity, total phenol content, and total flavonoid	[22]

Leaves (shade dried) (1 g)	Heat reflux	50% methanol	60°C + 1 h	Chlorogenic acid: 0.05% Quercetin: 0.02 Asiaticoside: 0.41% Madecassoside: 0.54 Asiatic acid: 1.69% Madecassic acid: 0.21 Rutin: 0.03	Antioxidant activity (IC _50_ = DPPH [96.28 *μ*g/mL]) ABTS (79.16 *μ*g/mL)	[24]
	Ultrasound	50% methanol	60°C	Chlorogenic acid: 0.11 Asiaticoside: 1.87% Asiatic acid: 1.49% Rutin: 0.05 Quercetin: 0.02 Madecassoside: 0.53 Madecassic acid: 0.11	Antioxidant activity (IC _50_ = DPPH [44.57 *μ*g/mL]) ABTS (79.16 *μ*g/mL)	
	Microwave	50% methanol	800 W power+2-min irradiation time+3 extraction cycles	Chlorogenic acid: 0.18% Quercetin: 0.02% Kaempferol: 0.10% Rutin: 0.11% Asiaticoside: 2.66% Asiatic acid: 1.98% Madecassoside: 0.76% Madecassic acid: 0.32%	Antioxidant activity (IC _50_ = DPPH [45.17 *μ*g/mL]) ABTS (26.38 *μ*g/mL)	

Leaves	Ultrasound	Betaine–levulinic acid–NADES	Time: 32 min; power: 140 W; temperature: 36°C and 49 mL/g L/S ratio	Asiaticoside: 229.92 ± 1.67 mg/g		[28]

Leaves	Ultrasound	80% ethanol	48°C + 50 min	Madecassoside: 2.262 ± 0.046% *w*/*w* Asiaticoside: 1.325 ± 0.062% *w*/*w* Madecassic acid: 0.082 ± 0.009*w*/*w* Asiatic acid: 0.052 ± 0.007% *w*/*w*		[29]

Leaves	Microwave	80% ethanol	100 W, 7.5 min	Madecassoside: 7.332 ± 0.386% *w*/*w* Asiaticoside: 4.560 ± 0.153% *w*/*w* Madecassic acid: 0.357 ± 0.013% *w*/*w* Asiatic acid: 0.209 ± 0.025% *w*/*w*	Antioxidant activity

Dried leaves (40°C + 18 h)	Microwave	Methanol (1:25) (solid to solvent)	600 W + 10 min	Madecassoside: 25.05 ± 0.19 mg/g.m Asiaticoside: 48.49 ± 0.64 Madecassic acid: 5.91 ± 0.97 Asiatic acid: 1.85 ± 0.47	Antioxidant activity	[22]

Dried leaves	Microwave	NADES+acetylcholine chloride:malic acid:water (1:2:2)	300 W + 15 min	Madecassoside: 21.7 mg g^−1^ dry weight Asiaticoside: 12.7 mg/g dry weight	Antioxidant activity (IC_50_ = 0.26 mg.m/L)	[30]

20 g powdered leaves	Solvent-free microwave extraction		300 W + 15 min	Asiaticoside: 158.8 *μ*g/mL		[31]

Dried leaves (20 g)	Solvent-free microwave		450 W + 40 min		Total phenolic content (2.39869 mg G/g)	[32]

Dried leaves	Supercritical fluid extraction	Methanol (10%)	10 MPa + 80°C	Asiaticoside: 0.8383%		[33]

Dried powder (3.6 kg)	Supercritical fluid extraction	Ethanol (95%)	35 MPa + 60°C + 3 h	Asiaticoside: 0.964 ± 0.005% Madecassoside: 0.655 ± 0.002% Asiatic acid: 0.576 ± 0.003% Madecassic acid: 0.426 ± 0.006% Terminolic acid: 0.307 ± 0.003%	Antioxidant activity (DPPH-37.52 ± 1.35), wound healing activity, MMP-2 inhibition activity, cell migration, and angiogenesis activity	[34]

**Table 2:** Effect of *Centella asiatica* extract on mechanical, barrier, and functional properties of the packaging film.

**Content of *Centella* extract**	**The material used for the preparation of the film**	**Mechanical**	**Properties barrier**	**Functional**	**Reference**
0% (without adding *Centella* extract)	4 g gelatin dissolved in distilled water through mechanical stirring and kept in water at 45°C(100%) + 30% glycerol	TS: 0.85 MPa EAB: 214.05%	WVP: 3.41 × 10^−5^ g.mm/h. m^2.^kPa	TPC: 3.06 mg/g of GAE DPPH: 31.21%	[54]
5% *Centella* is added to the packaging film	Gelatin+glycerol	TS: 0.98 MPa EAB: 216.52%	WVP: 3.38 × 10^−5^ g.mm/h. m^2.^kPa	TPC: 3.16 mg/g of GAE DPPH: 34.70%	
25% *Centella* extract	Gelatin+glycerol	TS: 2.15 MPa EAB: 334.04%	WVP: 1.93 × 10^−5^ g.mm/h. m^2.^kPa	TPC: 4.63 mg/g of GAE DPPH: 47.51%	

0% *Centella* extract	Gelatin film prepared from chicken skin 3 g in 50 mL distilled water+3 g carboxymethyl cellulose in 50 mL water+0.78 mL glycerol	TS: 3.0 × 10^−2^ MPa EAB: 223.05% Puncture test: 0.06 N	WVP: 1.03 g.m/s/p Light transparency: 0.82	TPC: 0.06 mL/g of GAE DPPH: 41.95% Reducing power: 0.48 nm	[55]

0.3% *Centella* extract	Chicken gelatin film+CMC+glycerol	TS: MPa: 5.0 × 10^−2^ MPa EAB: 281.00% Puncture test: 0.05 N	WVP: 1.11 g.m/s/p Light transparency: 0.86	TPC: 0.29 mL/g of GAE DPPH: 68.88% Reducing power: 0.66 nm	

0.7% *Centella* extract	Chicken gelatin film+CMC+glycerol	TS: MPa: 4.5 × 10^−2^ MPa EAB: 271.17% Puncture test: 0.06 N	WVP: 1.13 g.m/s/p Light transparency: 0.71	TPC: 0.36 mL/g of GAE DPPH: 89.26% Reducing power: 0.80 nm	

1 mL *Centella* extract	Rice starch gelatin+ZnOP+glycerol (30%)	TS: 3.49–4.63 MPa EAB: 92.20%–37.68%	WVP: 5.52 × 10^−11^–7.45 × 10^−11^ g.m/m^2^ s Light transmission (400–800 nm): 75.07–83.15 (control) and 0.33–18.82 for RS-G-ZnONP	Antibacterial: *Staphylococcus aureus*, *E. coli*, and *Salmonella typhimurium* Antifungal: *Aspergillus niger*	[56]

## Data Availability

Data sharing is not applicable to this article as no datasets were generated or analysed during the current study.
